# In this month’s Bulletin

**DOI:** 10.2471/BLT.25.000825

**Published:** 2025-08-01

In the editorial section, Ankur Rakesh et al. (466) call for action on extreme heat conditions experienced by populations worldwide. Abdullah A Al Rabeeah et al. (467) explain why better alignment of the humanitarian system is needed to address current operational gaps in conflict-affected areas. 

## Afghanistan, Angola, Burkina Faso, Central African Republic, Democratic Republic of the Congo, India, Mali, Nigeria, Pakistan, Somalia, South Sudan, Sudan, Syrian Arab Republic, West Bank and Gaza Strip

### Delivering polio vaccines

Kamran Badizadegan & Kimberly M Thompson (484–492) review reports of attacks on vaccination personnel.

## Bangladesh, Benin, Chad, China, Egypt,Ethiopia, Guinea, India, Kenya, Malawi, Nepal, Nigeria, the Philippines, Romania, Somalia, United Republic of Tanzania

### Pregnancy-associated acute kidney injury

Phu Nguyen Trong Tran et al. (493–506) establish incidence estimates.

## Pakistan

### Ending leprosy

Anil Fastenau et al. (507–514) propose a roadmap for elimination.

## Global 

### Breast cancer outcomes

Syed Mahfuz Al Hasan et al. (470–483) analyse the association between regular screening programmes and mortality reductions.

### Improving men’s health

Morna Cornell (515–516) argues that specific policies are long overdue.

